# Clinical profiles, epidemiological characteristics and treatment outcomes of COVID-19 patients in North-eastern Ethiopia: A retrospective cohort study

**DOI:** 10.1371/journal.pgph.0002285

**Published:** 2023-09-20

**Authors:** Alemu Gedefie, Tadesse Birara, Sisay Misganaw, Getachew Mesfin Bambo, Samuel Sahile Kebede, Mihret Tilahun, Ousman Mohammed, Yeshimebet Kassa, Habtye Bisetegn, Ermiyas Alemayehu

**Affiliations:** 1 Department of Medical Laboratory Sciences, College of Medicine and Health Sciences, Wollo University, Dessie, Ethiopia; 2 Department of Information System, College of Informatics, Wollo University, Kombolcha, Ethiopia; 3 Department of Medical Laboratory Sciences, College of Health Sciences, Mizan-Tepi University, Mizan, Ethiopia; PLOS: Public Library of Science, UNITED STATES

## Abstract

**Background:**

COVID-19 is a rapidly emerging global health threat and economic disaster. The epidemiology and outcomes of COVID-19 patients in Ethiopia are scarce. Thus, the present study aimed to assess clinical profiles, epidemiological characteristics, and treatment outcomes of patients with COVID-19 and to identify determinants of the disease outcome among COVID-19 patients in North-eastern Ethiopia.

**Methods:**

A retrospective observational cohort study was conducted in North-eastern Ethiopia, from May 2020 to Jan 2022 on a total of 364 SARS-COV-2 infected patients. Demographic and clinical data were abstracted from the medical records of patients. Bivariable and multivariable analyses were conducted to determine the factors associated with the mortality of COVID-19 patients and variables with a P-value < 0.05 were considered statistically significant.

**Result:**

Among 364 COVID-19 patients included in this study, two-thirds (68.1%) were males with a median age of 34 years. The majority; 42.9% & 33.0% respectively cases were detected at the health facility and community level surveillance. Furthermore, 6.6% of patients had pre-existing comorbidities of which diabetes mellitus (23.1%) and hypertension (15.3%) had the highest frequency. The symptomatic rate of COVID-19 patients was 30.5%. The most common clinical presentations were cough (26.9%), fever (26.1%), and shortness of breath (15.2%). Moreover, the mortality rate of COVID-19 patients was 4.1% which was independently predicted by a history of underlining co-morbidity (AHR:6.09; 95%CI:1.299–28.56; *P = 0*.*022*) and a history of severe or critical conditions (AHR 11.8; 95%CI:4.89–28.83; *P = 0*.*003*).

**Conclusion:**

Severe or critical acute COVID-19 and underlining comorbidities are associated with higher mortality. Therefore, critical follow–up and management should be given to patients with underlying diseases is required.

## 1. Introduction

Coronavirus disease 2019 (COVID-19) is a rapidly emerging global health threat that has been classified as a pandemic by the World Health Organization (WHO). The coronavirus disease (COVID-19) pandemic, caused by severe acute respiratory syndrome coronavirus 2 (SARS-CoV-2) [1], has devastated the world in the space of just a few months. Since it was first reported on December 31, 2019, in the Hubei province of China, in the mid of March 2023, over 760 million people have been infected globally with over 6.8 million deaths as of March 16, 2023, WHO report [1, 2]. At the regional level; the Western Pacific Region reported unique findings where the number of new weekly cases and deaths increased by 32% and 22% respectively in any of WHO regions including Africa [3]. Particularly in Ethiopia; over 500 thousand of cases have been confirmed and more than 7 thousand deaths were recorded [2].

SARS-CoV-2 incubation times might range from 2 to 14 days [4]. Nearly 80% of infected individuals exhibited quite minor symptoms or showed no symptoms at all. and 20% of those patients displayed serious symptoms that necessitated inpatient care [5] Although mild SARS-CoV-2 sickness did not necessitate hospitalization, the virus could still be spread in these instances [6]. SARS-COV-2 is transmitted through coughing and sneezing droplets. It can survive for up to six days at room temperature on contaminated surfaces and objects. The commonest symptoms at the onset of COVID-19 illness are dry cough, shortness of breath, fever, and fatigue, whereas other symptoms such as the production of sputum, diarrhoea, hemoptysis, dyspnea, headache, and lymphopenia [7–12]. After the development of sever clinical symptoms including pneumonia in the chest X-ray and usual symptoms are emerged between days 7 and 9 [8]. Finally, several co-morbidities such as cardiovascular and respiratory chronic diseases, diabetes, obesity, hypertension, and other diseases of the metabolism, have been implicated as risk factors for a poor prognosis [13–15].

The case fatality rate (CFR) among hospitalized patients was estimated to be roughly 5%, with an overall mortality rate of 0.25% an increased fatality rate with age, and patients with underlying disease [16]. Supportive care is the mainstay of treatment for COVID-19. Numerous antiviral medications, including chloroquine, hydroxychloroquine, lopinavir/ritonavir, remdesivir, and favipiravir, have been suggested for use in clinical trials due to the COVID-19’s new development and the lack of established treatment protocols [17–19].

About 89.4% of patients experienced clinical recovery, which took an average of 14 days to complete. Compared to data from other nations, especially those in Africa, the death rate of the population under study is lower. It was discovered that having a cough and having a severe COVID-19 illness severity were linked to a delayed time in clinical recovery. On the other side, hyperthermia is linked to a shorter course of disease (faster time to clinical recovery) [20]. Moreover, a poorer prognosis for the condition was linked to reduced oxygen saturation, subjective complaints of shortness of breath, and diabetes. For a better outcome, patients with these factors should be closely monitored [20].

The clinical characteristics of COVID-19 differ amongst populations, hence these characteristics may or may not be consistent with SARS-CoV-2 infected populations in Ethiopia. Despite this variation, only a small number of research have characterized the clinical traits of COVID-19 patients in Ethiopia [21]. There have been reports of various clinical presentations, disease courses, and outcomes that vary from study to study and region to region, suggesting the need for additional research to better understand the condition [20]. Understanding the epidemiological and clinical results of COVID-19 patients who are on treatment is critical for determining the efficacy of therapies and assessing service quality [22]. However, pieces of evidence on clinical outcomes and risk factors of COVID-19 patients admitted to hospitals in low-income countries, particularly Ethiopia are very scarce. Thus, this study aimed to assess the treatment outcomes as well as clinical profiles and epidemiological characteristics of hospitalized COVID-19 patients in the Northeastern parts of Ethiopia.

## 2. Materials and methods

### 2.1 Study area, design, period and population

This was a retrospective cohort study of SARS-COV-2 infected patients at COVID-19 treatment centers of Kemisse Hospital, Oromia Special Zone of Amhara, Ethiopia from May 2020 to Jan 2022. A total of 364 SARS-COV-2 infected patients who are on follow-up were included in the study (S1 Data). Chart review was carried-out from May 1 to May 10, 2022. Patients chart with missing data for socio-demographic, clinical, clinical and epidemiological variables were excluded from the study analysis. The study protocol and waiver of informed consent was approved by the Institutional Review Board of the College of Medicine and Health Sciences, Wollo University. Participants did not provide written informed consent for the use of their clinical records in this study; however, cases were de-identified and only code numbers were utilized the entire time.

### 2.2 Eligibility criteria

**Fig 1:**
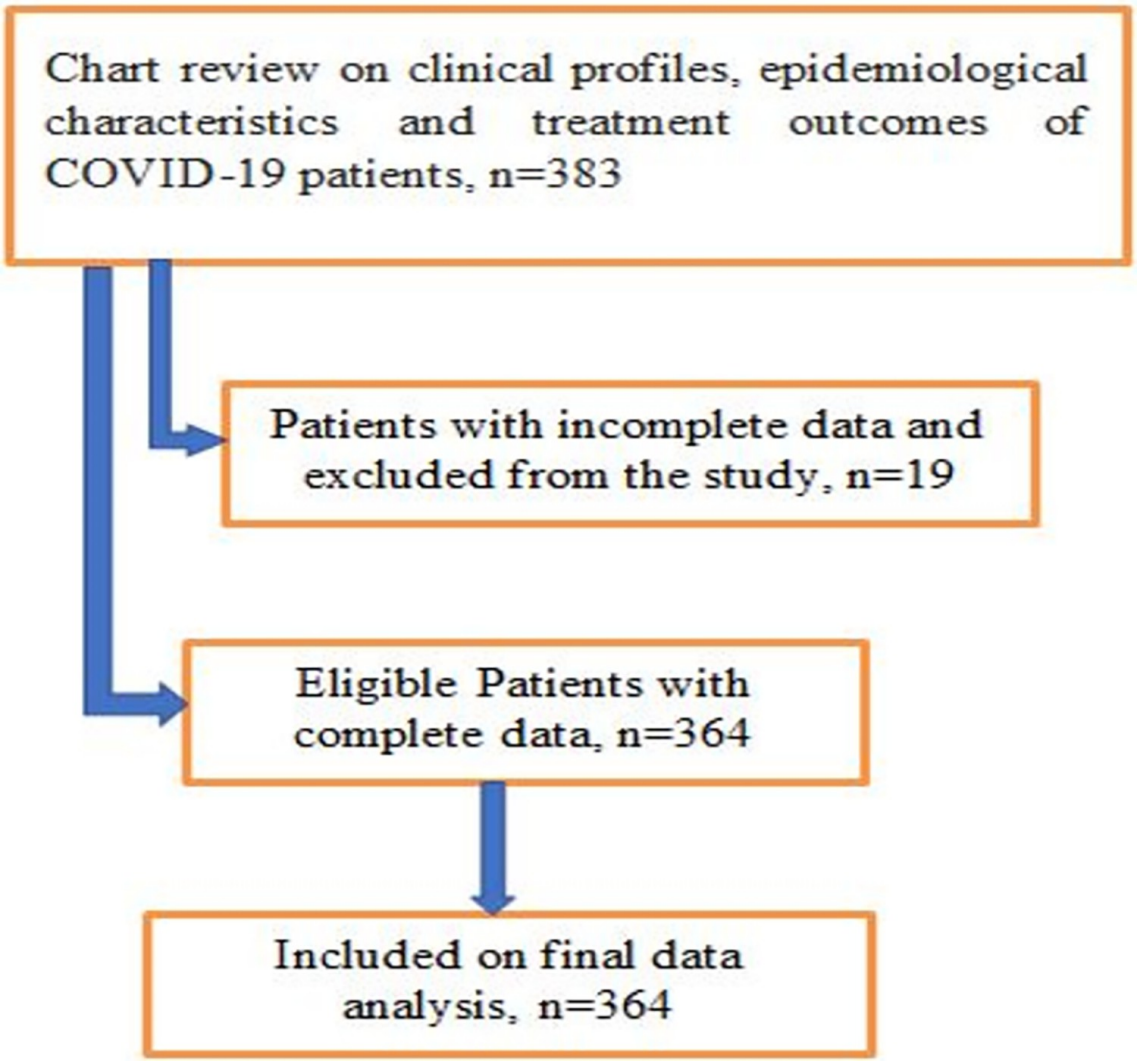
Enrolment procedure of COVID-19 patients in the study.

- All Patients with COVID-19 who were on treatment and follow-up at the center from May 2020 to Jan 2022 and with complete follow-up data were included (Fig 1).

### 2.3 Operational definition of COVID-19 disease [20]

- **Mild Disease:** characterized by fever, malaise, cough, upper respiratory symptoms, and/or less common features of COVID-19 (headache, loss of taste or smell etc…)
- **Moderate Disease:** Patients with lower respiratory symptom/s. They may have infiltrated on a chest X-ray. These patients can maintain oxygenation on room air.
- **Severe COVID-19 disease**: defined as patients with hypoxia: SPO2<93% on atmospheric air or PaO2:FiO2 < 300mmHg (SF ratio < 315); tachypnea: in respiratory distress or RR>30 breaths/minutes; and more than 50% involvement seen on chest imaging.
- **Clinical recovery**: implies resolution of symptoms and/ or signs of patients as evidenced by clinical, laboratory, and radiologic assessments irrespective of the biochemical recovery. Event: Clinical recovery from COVID-19.
- **Severe Malnutrition**: defined as adults with BMI of less than 18.5 kg/m^2^ and children with lower than the median minus 3 standard deviation of weight for height, weight for age and height for age scores.
- **Waves (W) of COVID**-19 were defined according to the figures of nationally reported COVID-19 cases (**W1**: March 2020 to November 2020; **W2**: December 2020 to June 2021; **W3**: July 2021 to January 2022) [2, 23].

### 2.4 Variables

COVID-19 treatment outcome was the dependent variable while socio-demographic, clinical and epidemiological variables were considered as explanatory variables

### 2.5 Data collection

Data were abstracted from the medical records of the patients retrospectively. The data obtained included demographics (e.g., age, sex, residence zone and woreda, occupation, site of detection), clinical profiles (fever, headache, dry cough, generalized body weakness, shortness of breath, sore throat, pain, and other symptoms); underline comorbidity and conditions; travel history in the last 14 days to high-risk areas and traveling country/area; Close contact with confirmed COVID-19 case; source of infection as well as the degree of severity and outcome status of infected patients.

### 2.6 Statistical analysis

Patient data were registered into Microsoft excel and analyzed using SPSS version 25 software (SPSS Inc., Chicago, IL, USA). Categorical variables were summarized by frequencies and percentages, and continuous variables were summarized by mean ± standard deviation (SD). Bivariable logistic regression analyses were performed to assess predicting factors associated with treatment outcomes of COVID-19. Variables that were found to be significant in bivariable analysis (P < 0.25) were included in the multivariable backward stepwise logistic regression model to identify factors independently associated with treatment outcomes of COVID-19 patients. Finally, variables with an adjusted hazard ratio with its P-values < 0.05 were considered significant.

## 3. Result

### 3.1 Socio-demographic characteristics

**Fig 2:**
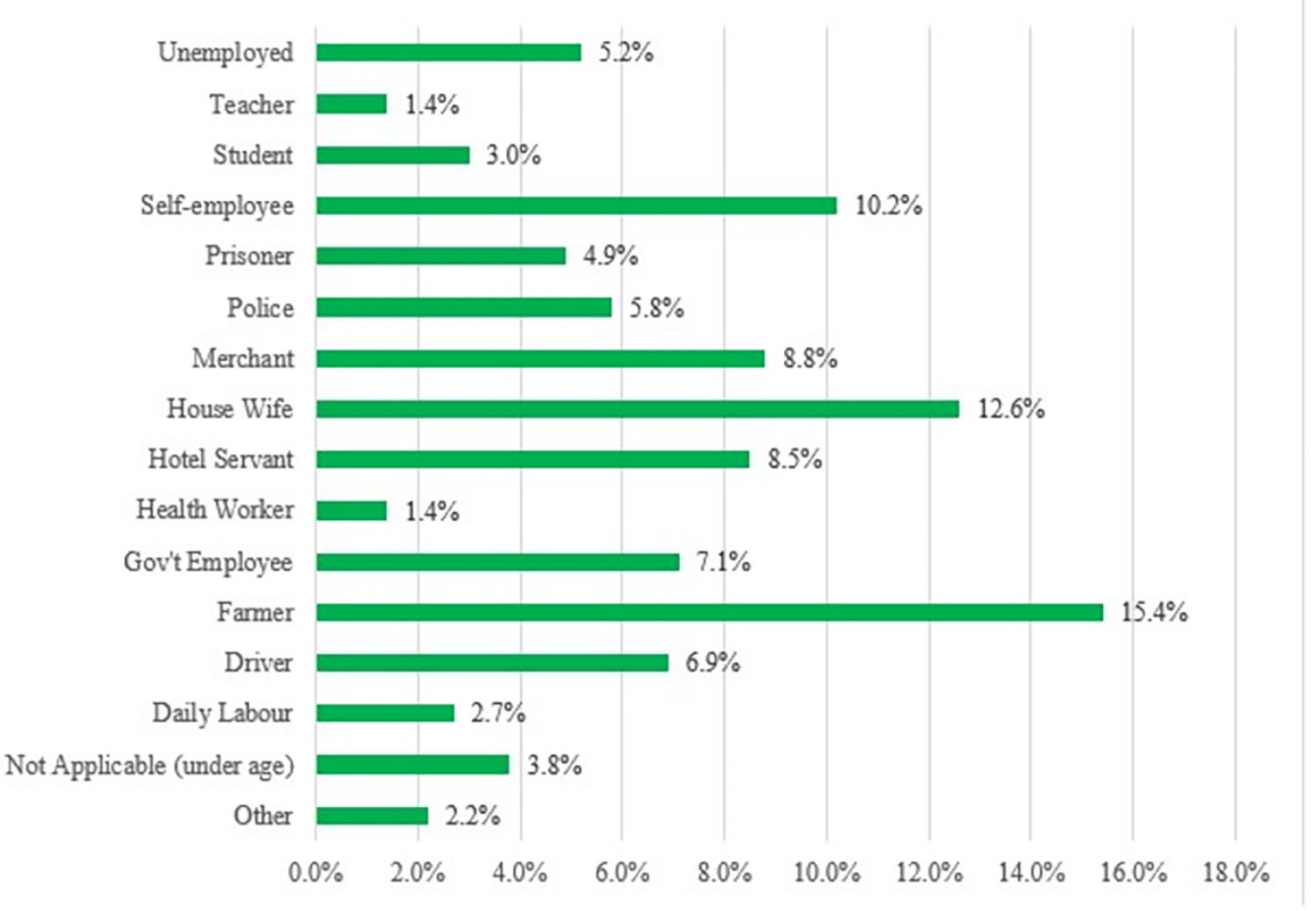
Occupational category of COVID-19 patients.

**Fig 3:**
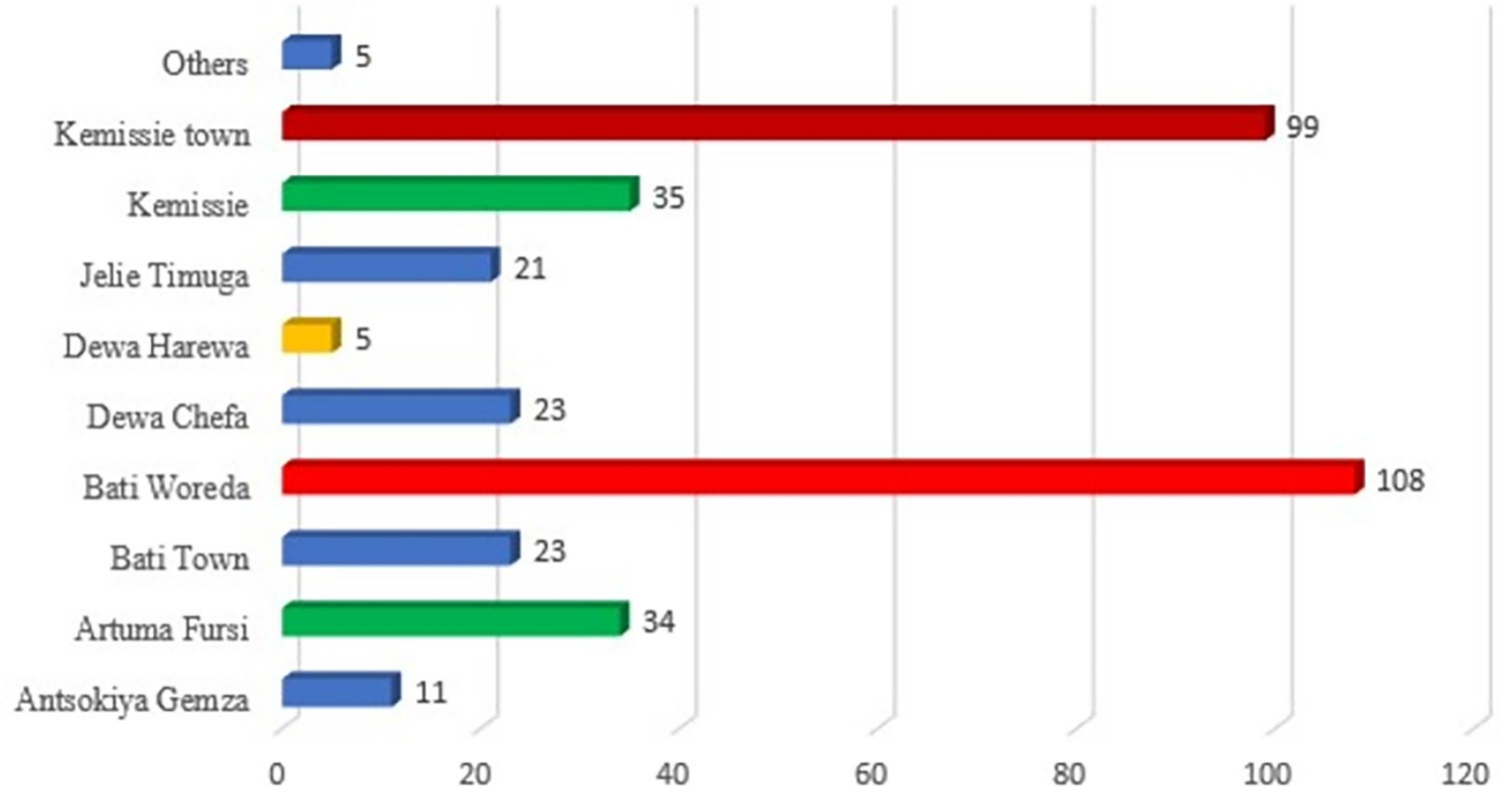
Distribution of COVID-19 cases by woreda of residence in North-east Ethiopia.

A total of 364 COVID-19 confirmed patients were included in the final analysis of the study. The median age of the patients was 34 years (IQR: 23–40), 28% of patients were older than 45, and about two-thirds (68.1%) of patients were males. All patients were from three zones of the Amhara region with majorities (96.4%) from Oromia Special Zone (Table 1). Moreover, 15.4% and 12.6% of patients were farmers and housewives respectively (Fig 2). Regarding, the area of residence majority of patients was found in Bati Woreda (29.7%) and Kemisse town (27.2%) respectively (Fig 3).

**Table 1:** Socio-demographic characteristics of COVID-19 confirmed patients in North-Eastern Ethiopia.

Variable	Category	Frequency; No (%)
Age group	Younger than 15	21(5.8)
	15–24	62(17.0)
	25–34	98(26.9)
	35–44	81(22.3)
	Older than 45	102(28.0)
Sex	Female	116(31.9)
	Male	248(68.1)
Residence by zone	North shewa	11(3.0)
	Oromia Special Zone	351(96.4)
	South Wollo	2(0.5)

### 3.2 Outbreak investigation, clinical profile and treatment outcomes

**Fig 4:**
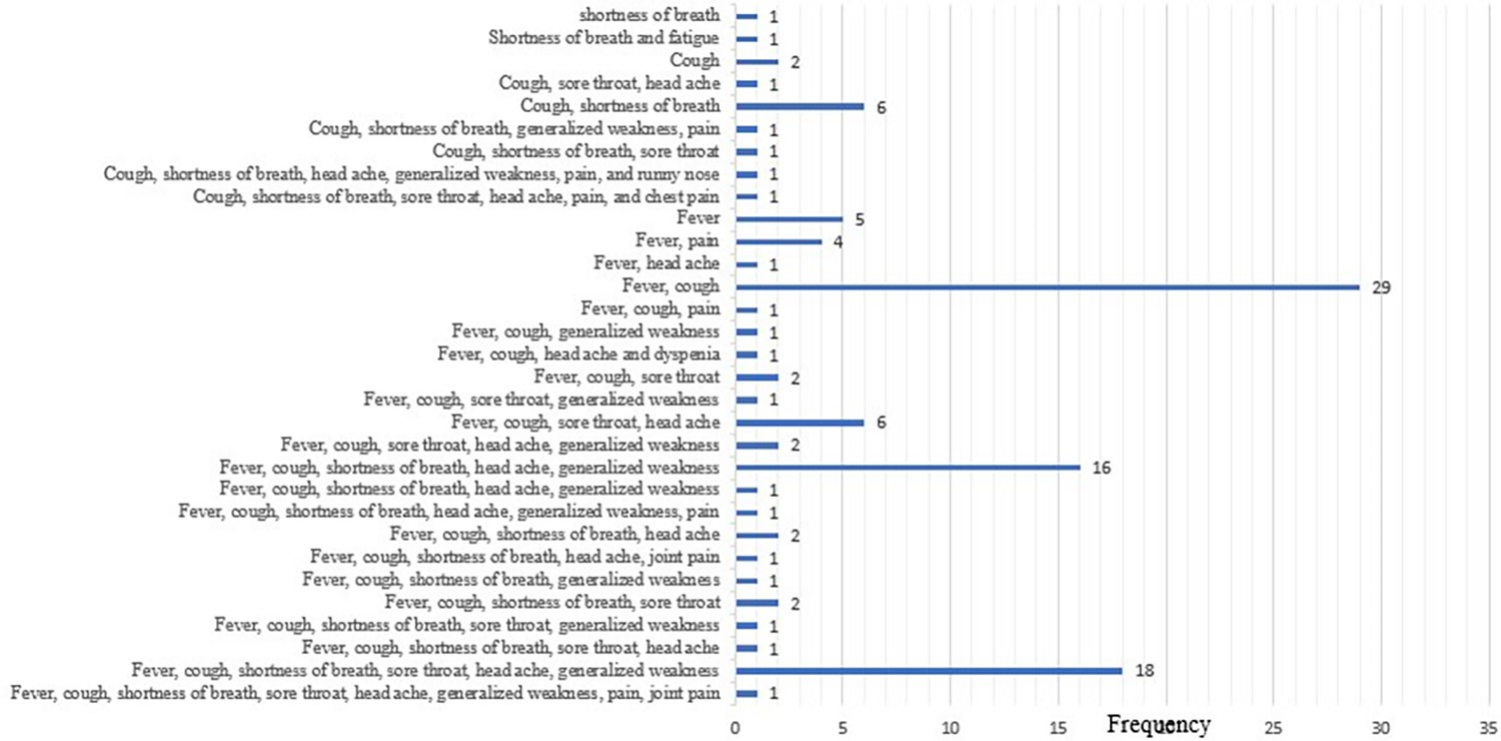
Frequency of spectrum of clinical characteristics of COVID-19 confirmed patients in North-eastern Ethiopia.

The majority of cases were detected at health facilities (42.9%) and community level surveillance (33.0%) and the least were from car inspection sites (0.5%). About 4.4% of patients had a travel history in the last 14 days before they were confirmed to have COVID-19, 6.6% of patients had pre-existing medical conditions, of which diabetes mellitus (DM) (n = 4; 16.7%), sever acute malnutrition (n = 3; 12.5%) and asthma (n = 3; 12.5%) were the most common comorbidities, in addition, two patients had both hypertension and DM. About 20.1% of patients had close contact with a confirmed case and were considered as a source of infection. Nearly one-third (111; 30.5%) of patients were symptomatic, of which cough (n = 101; 26.9%), fever (n = 98; 26.1%), and shortness of breath (n = 57; 15.2%) were the most frequently reported symptoms (Table 2). A single presenting symptom had reported in 2.2% of patients whereas 97.8% had two or more presenting symptoms (Fig 4). Furthermore, the mortality rate of COVID-19 patients was 4.1% (15/364) as indicated in Table 2.

**Table 2:** Clinical profile; outbreak investigation characteristics and treatment outcomes of COVID-19 confirmed patients in North-Eastern Ethiopia.

Variables	Category	Frequency (%)
Site of case detection	Bus Station	3(0.8)
	Car Inspection Site	2(0.5)
	Community	120(33.0)
	Health Facility	156(42.9)
	Home detection	10(2.7)
	Military Barracks	16(4.4)
	Prison	18(4.9)
	Quarantine center	32(8.8)
	Treatment Center	7(1.9)
Travel History in the last 14 days	No	348(95.6)
	Yes	16 (4.4)
Travelling country	Djibouti	13(3.6)
	Somalia	3(0.8)
Close contact with confirmed case	No	3(0.8)
	Unknown	288(79.1)
	Yes	73(20.1)
Exposure history	Contact history	73(20.1)
	Not identified	276(75.8)
	Travel History	15(4.1)
Underline Comorbidity and conditions	No	340(93.4)
	Yes	24(6.6)
Comorbidities type (n = 24)	Asthma	3(12.5)
	Congested heart failure	2(8.3)
	Diabetes Mellitus	4(16.7)
	Sever acute malnutrition	3(12.5)
	HIV	1(4.2)
	Hypertension	2(8.3)
	Liver Disease	2(8.3)
	Pregnancy	2(8.3)
	Pulmonary Tuberculosis	1(4.2)
	Chronic respiratory disease	1(4.2)
	Sever acute malnutrition & severe anaemia	1(4.2)
	Diabetes Mellitus & Hypertension	2(8.3)
Symptomatic state	No	253(69.5)
	Yes	111(30.5)
Clinical sign and symptoms (n = 111)	Fever	98(26.1)
	Cough	101(26.9)
	Shortness of breath	57(15.2)
	Sore throat	36(9.6)
	Head ache	38(10.1)
	Generalized weakness	29(7.7)
	Pain	10(2.7)
	Other symptoms	6(1.6)
Severity level of symptomatic patients (n = 111)	Mild	47(42.3)
	Moderate	43(38.7)
	Severe	21(18.9)
Treatment Outcomes of COVID-19 Confirmed patients	Death	15(4.1)
	Recovered	349(95.9)

**Fig 5:**
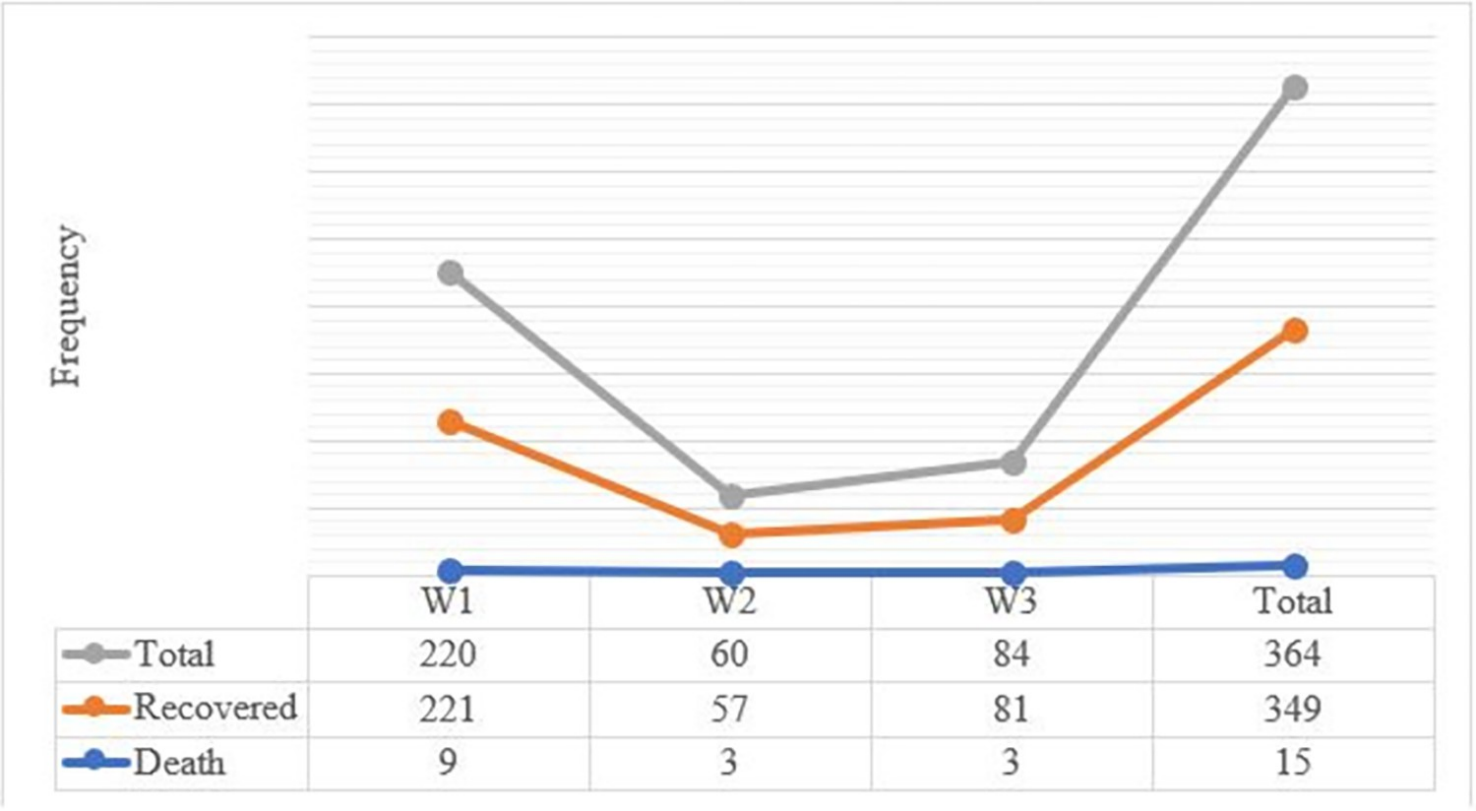
Patterns of treatment outcome across the waves of COVID-19 in Northeast Ethiopia.

A total of 220 (60.4%) of COVID-19 patients were reported in the first wave of Ethiopia. Moreover, the incidence rate of mortality due to COVID-19 was 5% (3/60) followed by 4.1% (9/220) in the second and the first waves respectively (Fig 5).

### 3.3 Predictors of COVID-19 treatment outcome

Most of the clinical profiles were found associated with COVID-19 bad treatment outcomes in bivariable logistic regression while after adjusting the confounding variables in the multivariable logistic regression model history of underlining co-morbidity (*P = 0*.*022*) and history of severe or critical conditions (*P = 0*.*003*) were an independent predictor of COVID-19 treatment outcome as shown in Table 3.

**Table 3:** Factors associated with treatment outcomes of COVID-19 confirmed patients in North-Eastern Ethiopia.

Variables	Category	Treatment Outcome		CHR (95% CI)	P-value	AHR (95% CI)	P-value
		Death; N (%)	Recovery; N (%)				
Gender	Male	13(5.2)	235(94.8)	2.317(1.07–3.49)	0.135	3.02(1.04–4.128)	0.070
	Female	2(1.7)	114(98.3)	1		1	
Sore throat	Yes	4(11.1)	32(88.9)	3.6(1.08–11.96)	0.036*	3.01(0.26–34.95)	0.378
	No	11(3.4)	317(96.6)	1		1	
Head ache	Yes	4(10.5)	34(89.5)	3.36(1.01–11.1)	0.047*	0.79(0.067–9.29)	0.852
	No	11(3.4)	315(96.6)	1		1	
Generalized weakness	Yes	4(13.8)	25(86.2)	4.7(1.39–15.87)	0.012*	1.17(0.104–13.11)	0.900
	No	11(3.3)	324(96.7)	1		1	
Underline comorbidity	Yes	4(16.7)	20(83.3)	5.98(1.75–20.4)	0.004*	**6.09(1.299–28.56)**	0.022*
	No	11(3.2)	329(96.8)	1		1	
Shortness of breath	Yes	6(10.5)	51(89.5)	3.89(1.33–11.4)	0.013*	3.53(0.388–32.18)	0.263
	No	9(2.9)	298(97.1)	1		1	
Fever	Yes	5(5.1)	93(94.9)	1.37(0.45–4.13)	0.569		
	No	10(3.8)	256(96.2)	1			
Cough	Yes	6(5.9)	95(94.1)	1.78(0.61–5.14)	0.285		
	No	9(3.4)	254(96.6)	1			
Pain	Yes	1(10.0)	9(90.0)	2.69(0.31–22.79)	0.362		
	No	14 (4.0)	340(96.0)	1			
Symptomatic state	Yes	7(6.3)	104(93.7)	2.06(0.73–5.89)	0.173	0.39(0.045–3.47)	0.402
	No	8(3.2)	245(96.8)	1		1	
Severity status	No symptoms/ mild	8(2.7)	292(97.3)	1		1	
	Moderate	1(2.3)	42(97.7)	0.896(0.14–9.42)	0.15	-	-
	Severe/ Critical	6(28.6)	15(71.4)	14.6(4.49–47.45)	0.000	11.8(4.89–28.83)	0.003*

**CHR**: Crude Hazard Ratio; **AHR**: Adjusted Hazard Ratio; *: Statistically significant; **CI**: Confidence Interval; **N**: Number; **%**: Percent

## 4. Discussion

Over time, a large number of SARS-CoV-2 variants have been identified that exhibit genetic variations as a result of spontaneous mutations. Majority of these mutations causes SARS-CoV-2 to adapt better to its hosts. The variants of public health concern identified after the original strain sequenced on 07^th^ January 2020 to 2022 were alpha, beta, gamma, delta and omicron. Of these variants, beta, delta and omicron were predominantly distributed in African countries including Ethiopia. Both the disease’s progression and its outcome vary depending on these mutations [24–26]. For this reason, there have been reports of various clinical presentations, disease courses, and outcomes that vary from study to study and region to region, suggesting the need for additional research to better understand the condition. Globally, COVID-19 evidence is being produced quickly aimed at understanding the clinical profile and better management of the disease. Thus, various sociodemographic and clinical factors, which may vary by region, determine the severity and mortality of COVID-19. The data supporting African COVID-19, however, is scant. Thus, this retrospective cohort study was conducted to summarize the socio-demographics, clinical characteristics, and level of severity of mild to severe COVID-19 patients and their outcome status in the North-eastern parts of Ethiopia. In this study, about two-thirds (68.1%) of the study population were males because of women’s stronger innate and adaptive immune systems, and women are believed to be less likely than men to be harmed by numerous germs and viruses [27]. Thus, being male is considered a risk factor for SARS-COV-2. Furthermore, this study was done on all age groups with the majority (28.0%) of participants were found above 55 years of age. Health facilities (42.9%) followed by community level surveillance (33.0%) were the predominant area of outbreak investigations in the study area.

About 6.6% of participants had underline comorbidity and/or medical conditions which will worsen the pathogenesis of COVID-19. This proportion was consistent with 5.1% reported from a study of Northern Ethiopia [21] but lower than 29% of comorbidity reported from a study of Thailand [28] which might be due to differences in the target population’s socio-demographic characteristics and health status. The most frequent underlying comorbidities were Diabetes Mellitus (23.1%) followed by hypertension (15.3%) which was comparatively consistent with a result found in the meta-analysis in China [29]. Likewise, the state of predominance was consistent with a study of Thailand [28]. However, the current finding was higher than previous findings reporting 1.6% of hypertension and 1.1% of diabetes mellitus from Woldia, Northeast Ethiopia [21] and significantly lower than study in Saudi Arabia [30] where 31% and 24% diabetes mellitus and hypertension respectively were reported. The existing discrepancies could be due to variations in lifestyles of the populations where both comorbidities are highly determined by personal lifestyles.

The symptomatic rate of SARS-COV-2 infected patients was 30.5% which was lower than the 94.4% symptomatic rate of COVID-19 in Thailand [28] and 77% from the study of Saudi Arabia [27]). The existing difference could be variations between target populations, age group, sex, the presence of underlying diseases [31], and other risk factors acquisition of SARS-COV-2 infected patients. The most frequent presenting symptoms in this study were cough (26.9%), followed by fever (26.1%), and shortness of breath (15.2%). The predominance of these clinical profiles was consistent with a study of Northeast Ethiopia [21] and Saudi Arabia [30]. Despite these being the most common factors, another spectrum of clinical manifestations has been reported in the present study (Fig 3). Among symptomatic COVID-19 patients, mild to severe cases were reported in the present study with a different proportion. Thus, 42.3%, 38.7%, and 18.9% of symptomatic COVID-19 patients respectively were mild, moderate, and severe. The proportion of severe COVID-19 patients in this study was comparable with the 15.7% proportion reported in a study in China [32] and 18% in Thailand [28].

The frequency of mortality rate in this study was 4.1% which was consistent with the death rate reported in Saudi Arabia, 4% [30]. Moreover, the proportion revealed in this study disagreed with a study in Thailand where no mortality was reported [28] and the previous 1.9% of mortality rate in Ethiopia [33]. These differences could be the result of differences in health care quality, sample size, age, gender, critical/severe illness of patients as well as underlining comorbidities, such as diabetes mellitus, hypertension, asthma, chronic pulmonary disease (COPD), Immune status, HIV, etc all of which might worsen the disease progression and mortality rate.

In this study, COVID-19 patients with underlying comorbidities (AHR:6.09; 95%CI:1.299–28.56; P = 0.0022) were 28.56 times at risk of COVID-19 mortality rate compared with their counterparts which were consistent with the Southern Ethiopian study which revealed that COPD, DM, asthma, HIV infection, and chronic kidney disease (CKD) were the most common predicting factors [34]. Furthermore, patients with severe/critical conditions were increased by 11.8 times at risk of COVID-19 associated mortality rate (AHR 11.8; 95%CI:4.89–28.83; P = 0.003) than those patients with no symptoms / mild symptoms. This finding was supported by findings from China [35] and India [36] where poor treatment outcome was found in patients with critical or severe conditions due to worsening health conditions, especially in patients with other underlining conditions or chronic illness. The proportion of mortality rate of COVID-19 patients was 3 times higher in males than in females (P>0.05). This might be due to immunologic differences. When compared to males, females exhibit a more robust antibody response to COVID-19 and recover more quickly. This is because X inactivation promotes a strong immunological response in females. Poor outcomes and an inflammatory response that is dysregulated in some males with COVID-19 are also explained by low testosterone levels [37]. As limitations, multiple clinical variables associated with in-hospital mortality were not assessed such as COPD, CKD, Antiviral therapies, vaccination, reinfection, type of VOC infection.

## 5. Conclusion and recommendation

The majority of COVID-19 patients included in this study were males. Health facility and community level investigations were the most common outbreak investigation sites used for the detection of COVID-19. One-third of COVID-19 patients were symptomatic and cough, fever, and shortness of breath were the most frequently reported symptoms in the study. Thus, an increased rate of asymptomatic infection found in this study could be a sign of community-wide asymptomatic transmissions. History of underlining comorbidities and severe/critical conditions were significantly associated with a higher rate of mortality among COVID-19 patients. Therefore, critical follow–up and management of patients with underlying diseases and worsening health conditions during admission is highly required. We also found that the

## Supporting information

S1 Data COVID-19 treatment outcome data set. (XLSX)
